# ST-elevation myocardial infarction following de-escalation to aspirin monotherapy: evidence of residual platelet function MRP4 overexpression dependent case report

**DOI:** 10.1093/ehjcr/ytag385

**Published:** 2026-05-22

**Authors:** Vincenzo Cesario, Eleonora Schiera, Mariaignazia Curreli, Giuliano Tocci, Fabio Maria Pulcinelli

**Affiliations:** Cardiology Unit, Cardiothoracic-Vascular and Heart Transplant Department, San Camillo Forlanini Hospital, Circonvallazione Gianicolense 87, Rome 00152, Italy; Department of Experimental Medicine, Sapienza University of Rome, Viale Regina Elena 324, Rome 00161, Italy; Department of Experimental Medicine, Sapienza University of Rome, Viale Regina Elena 324, Rome 00161, Italy; Department of Clinical and Molecular Medicine, Sapienza University of Rome, Sant′Andrea Hospital, Via di Grottarossa 1035-1039, Rome 00189, Italy; Department of Experimental Medicine, Sapienza University of Rome, Viale Regina Elena 324, Rome 00161, Italy

**Keywords:** Acute coronary syndrome, Aspirin, MRP4, Platelet reactivity, Antiplatelet therapy de-escalation, Case report

## Abstract

**Background:**

De-escalation from dual antiplatelet therapy (DAPT) to aspirin monotherapy is commonly performed after percutaneous coronary intervention to reduce bleeding risk. However, interindividual variability in platelet response exists, and high-on-aspirin residual platelet reactivity may predispose to thrombotic events. Platelet overexpression of multidrug resistance protein 4 (MRP4) has been proposed as a molecular mechanism contributing to aspirin low response, and it is a further risk factor for cardiovascular events.

**Case summary:**

An 82-year-old woman with a history of coronary artery disease presented with ST-elevation myocardial infarction 10 days after de-escalation from dual antiplatelet therapy (aspirin plus clopidogrel) to aspirin monotherapy. Coronary angiography revealed thrombotic occlusion of the proximal left anterior descending artery with in-stent restenosis. Platelet molecular analysis demonstrated increased MRP4 expression compared with aspirin-treated patients without acute coronary syndromes, suggesting impaired aspirin responsiveness consistent with a high-on-aspirin residual platelet reactivity phenotype. The patient was successfully treated with ticagrelor-based antiplatelet therapy and experienced no recurrent ischaemic or bleeding events during follow-up.

**Discussion:**

This case suggests that MRP4-mediated aspirin residual platelet function may contribute to thrombotic events following de-escalation to aspirin monotherapy. Assessment of platelet reactivity and molecular markers such as MRP4 expression, before switch from dual to single therapy, may help identify individuals at higher risk of aspirin failure and support a personalized approach to antiplatelet therapy.

Learning points

- De-escalation from dual antiplatelet therapy to aspirin alone may leave selected high-risk patients exposed to residual thrombotic risk.
- Platelet MRP4 overexpression is linked to high-on-aspirin residual platelet reactivity and his molecular profiling may support personalized antiplatelet strategies after PCI preventing aspirin failure.

## Introduction

Aspirin remains a cornerstone in antiplatelet therapy for patients at high cardiovascular (CV) risk. When combined with a P2Y12 inhibitor, it constitutes standard therapy after percutaneous coronary intervention (PCI), particularly in acute coronary syndromes (ACS) patients, typically for 12 months. Current guidelines increasingly support personalized DAPT durations based on ischaemic and bleeding risks.^1,2^

However, a significant interindividual variability exists in platelet responsiveness to antiplatelet agents, especially in women.^3,4^ A subset of patients exhibits high-on-aspirin residual platelet reactivity (HARPR), despite adherence to therapy.^5^ MRP4, a unidirectional transporter encoded by ABCC4, is upregulated in platelets upon chronic aspirin exposure, linked to reduced intracellular aspirin bioavailability and impaired antiplatelet effect impaired aspirin responsiveness.^6,7^

We report a case of ACS occurring shortly after DAPT withdrawal, with evidence of increased platelet MRP4 expression, supporting a potential role of this mechanism in aspirin resistance with relevant clinical implications.

## Summary figure

**Figure:**
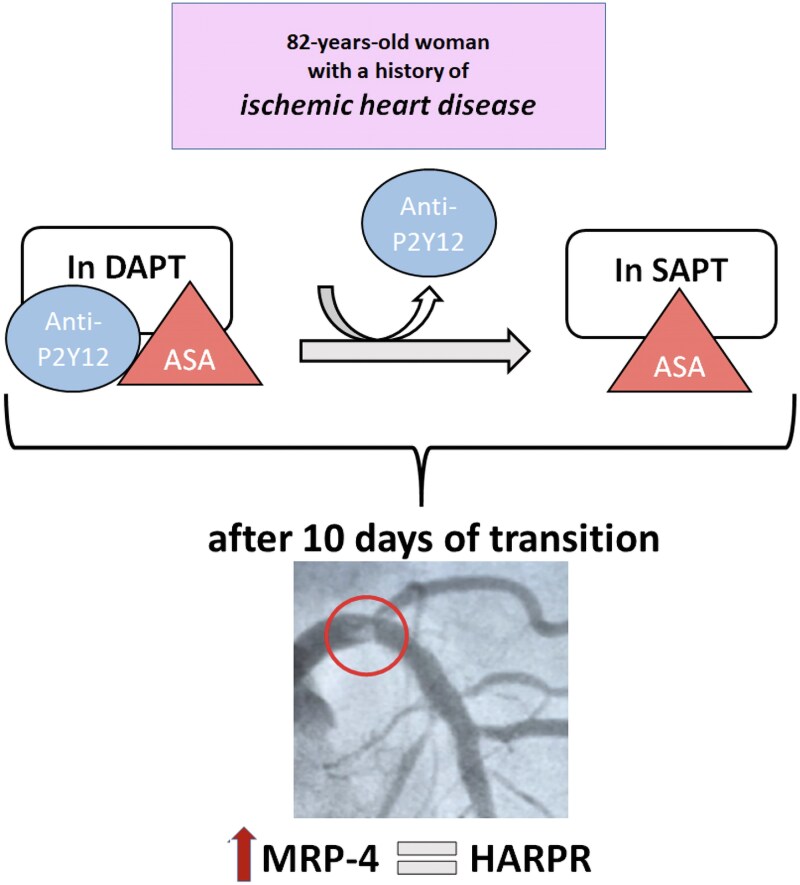
Timeline illustrating antiplatelet therapy de-escalation from dual antiplatelet therapy to aspirin monotherapy with a subsequent ST-elevation myocardial infarction and identification of increased platelet MRP4 expression leading to high-on-aspirin residual platelet reactivity.

## Case presentation

An 82-year-old woman with chronic coronary syndrome presented to the emergency department with acute chest pain. She had undergone PCI with drug-eluting stent (DES) placement in the left anterior descending (LAD) artery 1 year earlier for non-ST-elevation myocardial infarction (NSTEMI).

**Figure 1:**
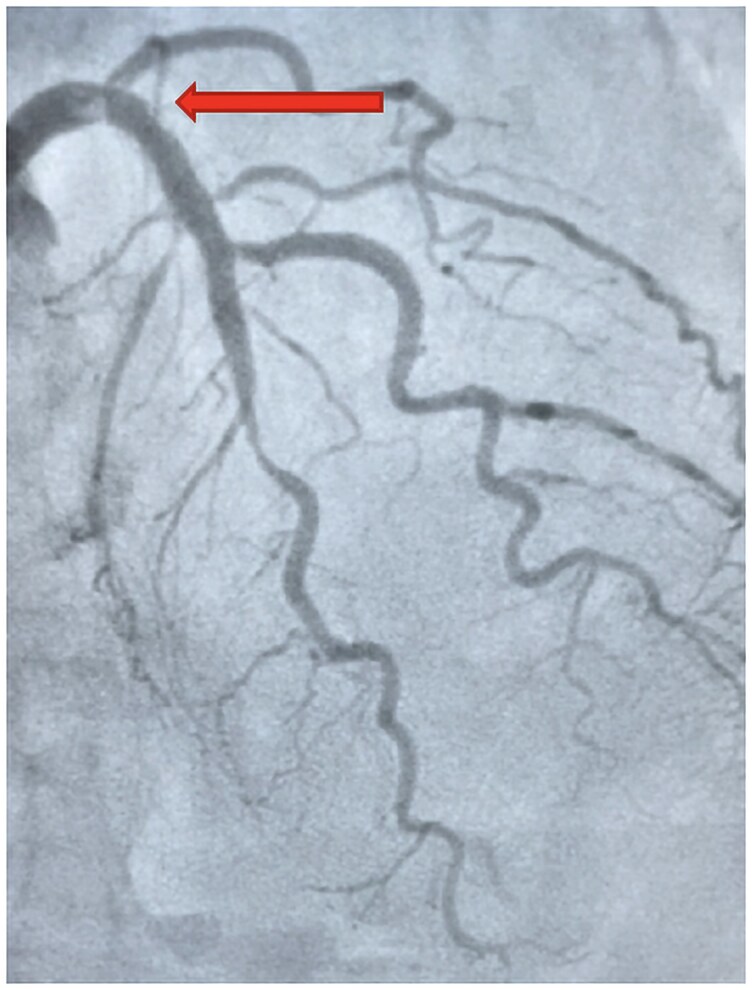
Coronary angiography showing a thrombotic occlusion (arrow) on the proximal segment of left anterior descending artery (LAD). This view shows the thrombus on the proximal descending coronary artery (LAD) and the in-stent restenosis on the mid LAD. RAO 10°CRA 40° view. CRA, cranial; RAO, right anterior oblique.

Emergency evaluation confirmed ST-elevation myocardial infarction (STEMI). Coronary angiography revealed a thrombus in the proximal LAD and in-stent restenosis in the mid-segment of the same vessel (*Figure 1*). Notably, 10 days prior, the patient had discontinued clopidogrel after 12 uneventful months of DAPT, continuing on aspirin monotherapy. ‘No concomitant comorbidities or medication, known to interfere with aspirin effect, were present at the time of event’.

**Figure 2:**
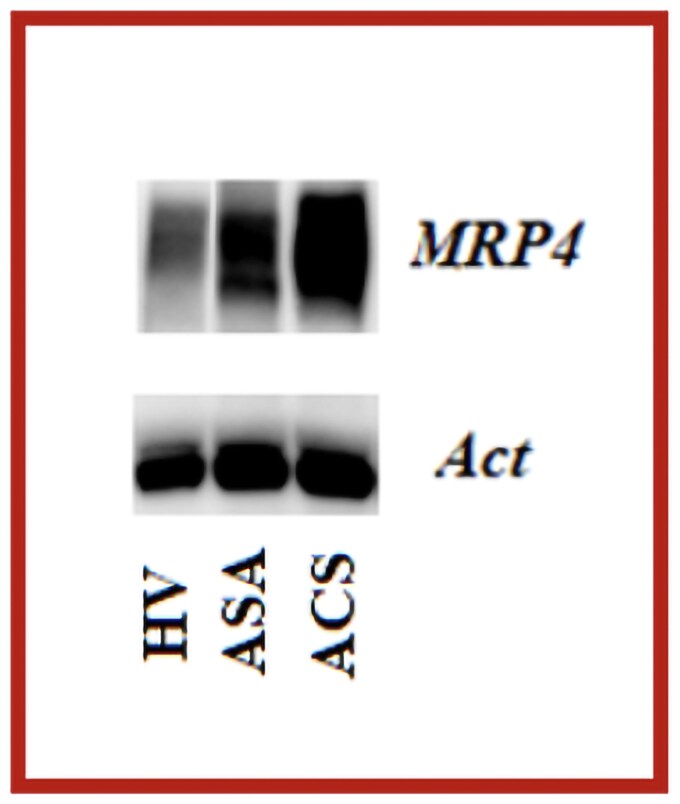
Representative western blot analysis of platelet multidrug resistance protein-4 (MRP4) expression in healthy volunteer (HV), in patient under chronic aspirin treatment without acute coronary syndrome and in patient under chronic aspirin treatment with acute ST segment elevation myocardial infarction (ACS).

Given the timing, a possible link between clopidogrel cessation and the acute event was suspected. Platelet function test could not be performed because the patient was administered 1 g of intravenous aspirin according to guidelines. During hospitalization, we analysed MRP4 expression in platelets via western blot. Platelet MRP4 expression was reported as the densitometric values (grey scale ratio between MRP4 and actin, housekeeping protein) for each patient evaluated as the fold increase compared to Healthy Volunteer (HV). The results show a 1.66-fold increase for ASA patient and 2.90-fold for ACS patient (*Figure 2*). These findings are consistent with prior studies associating MRP4 overexpression with HARPR.^5,8^ The higher MRP4 platelet expression that is 1.75 times higher in such patient compared to aspirin-treated patient without ACS strongly indicate an insufficient aspirin action after de-escalation.

The patient was treated with ticagrelor plus aspirin for 1 month, followed by ticagrelor monotherapy (90 mg b.i.d.) for 12 months. She experienced no bleeding or thrombotic events during follow-up. Subsequently, ticagrelor was de-escalated to 60 mg b.i.d.

## Discussion

This case report suggests that in ACS patients the transition from DAPT to aspirin monotherapy may have been insufficient for thrombotic protection for elevated platelet MRP4 expression. MRP4 overexpression is linked to HARRP,^8^ a prothrombotic phenotype associated with increased CV risk under aspirin treatment.^5^

Current guidelines recommend standard DAPT durations but increasingly recognize the need for personalization based on clinical and procedural risk.^9^ Studies such as GLOBAL LEADERS and TWILIGHT have demonstrated the efficacy of P2Y12 monotherapy after DAPT.^10,11^ Notably, ticagrelor monotherapy provided superior ischaemic protection compared to aspirin alone, albeit with a slightly increased bleeding risk.^11^ Evidence from randomized trials suggests that P2Y12 inhibitor monotherapy after an initial period of dual antiplatelet therapy may maintain ischaemic protection while reducing bleeding risk in selected patients. Our data demonstrate that a tailored strategy also for aspirin treatment whether via guided de-escalation can enhance safety while maintaining adequate antithrombotic coverage after PCI for acute coronary syndromes.^12^

Although platelet function testing and genetic assays are mentioned in ESC guidelines, they are not routinely employed in clinical practice. The importance of identifying platelet phenotype through specific biomarkers has been emphasized as a strategy to reduce residual thrombotic risk.^13^ In particular, optimizing the efficacy of antiplatelet requires a full understanding of the mechanisms responsible for treatment failure, thereby to address targeted therapeutic changes aimed at preventing recurrent events.

This case report suggests that molecular platelet profiling, including assessment of MRP4 expression, may help identify patients in whom aspirin monotherapy is insufficient and guide individualized antiplatelet strategies after PCI.^14^ In this context, recent transcriptomic and proteomic analyses have further highlighted the complexity of platelet biology and the role of gene expression profiles in modulating platelet function and haemostatic traits MRP4 dependent.^15^ Ideally, if platelet function and MRP4 expression had been assessed prior to DAPT discontinuation, a different de-escalation strategy—such as clopidogrel or ticagrelor monotherapy—might have been selected, potentially avoiding the recurrent STEMI. However, the present findings do not allow definitive causal inference, and MRP4 overexpression should be interpreted as a potential modulator of platelet function within a multifactorial thrombotic context. Larger prospective and case-control studies are needed to better define the clinical role of MRP4 in antiplatelet therapy personalization.

## Patient perspective

This case report suggests that aspirin-mediated MRP4 platelet over-expression could represent a potential contributing factor among a multifactorial thrombotic process to ACS event following DAPT de-escalation. These findings should be considered hypothesis-generating and warrant further investigation. Incorporation of platelet functional and molecular assessment, as MRP4 expression analysis, into clinical decision-making could be considered as a valuable tool in optimizing antiplatelet therapy, paving the way towards more individualized treatment strategies in order to assess the residual CV risk, for integrating a large amount of information with clinically actionable recommendations.^13^

## Data Availability

The data underlying this article are available from the corresponding author on reasonable request.

## Lead author biography

Dr. Vincenzo Cesario, MD, PhD, is an interventional cardiologist currently working at San Camillo Forlanini Hospital in Rome. He completed his PhD in Vascular Biology and Arterial Hypertension at Sapienza University of Rome and specialized in Cardiology with honors. After his specialization he has completed an Interventional Cardiology Fellowship program at Clinique Pasteur, Toulouse, France. He is a recipient of the EAPCI Andersen & Cribier Grant. Dr. Cesario has served as faculty at major international meetings, including EuroPCR, and has authored multiple peer-reviewed publications in interventional cardiology and structural heart disease.

## Notes

**Consent:** The authors confirm that written consent for submission and publication of this case report including image(s) and associated text has been obtained from the patient in line with COPE guidance.
